# The Effect of Inflammatory Markers on the Survival of Advanced Gastric Cancer Patients Who Underwent Anti-Programmed Death 1 Therapy

**DOI:** 10.3389/fonc.2022.783197

**Published:** 2022-02-01

**Authors:** Ziting Qu, Qianling Wang, Hui Wang, Yang Jiao, Min Li, Wei Wei, Yu Lei, Zhiyan Zhao, Tengteng Zhang, Yiyin Zhang, Kangsheng Gu

**Affiliations:** Department of Oncology, The First Affiliated Hospital of Anhui Medical University, Hefei, China

**Keywords:** stomach neoplasm, PD-1, inflammatory markers, prognosis, immunotherapy

## Abstract

**Purpose:**

This clinical study sought to determine whether the levels of inflammatory markers predicted the survival of advanced gastric cancer (AGC) patients who underwent anti-programmed death 1 (PD-1) therapy.

**Methods:**

Using AGC patient plasma samples and baseline characteristics, we investigated the specific value of inflammatory markers in AGC from a clinical perspective in immunotherapy.

**Results:**

One hundred and six patients with AGC who underwent anti-PD-1 therapy were enrolled in this study between 20 July 2019 and 16 March 2021. A significant decrease in NLR, dNLR, and SII was noticed among the PR (P=0.023; P=0.036; P=0.001), SD (P=0.048; P=0.022; P=0.023), ORR (P=0.021; P=0.032; P=0.001), and DCR (P=0.003; P=0.001; P<0.001) groups after anti-PD-1 therapy. Additionally, a significant decline of PLR was also observed in PR (P=0.010), ORR (P=0.007), and DCR (P=0.005) after anti-PD-1 therapy. Only MLR levels increased significantly at the time of anti-PD-1 immunotherapy the failure compared to baseline (P=0.039). And statistically significant elevations in NLR (P=0.001), MLR (P=0.020), dNLR (P=0.002), and SII (P=0.019) were found in failure of anti-PD-1 treatment compared to optimal efficacy in AGC patients. In first-line treatment, the number of metastatic sites (P=0.001) was an independent prognostic factor for PFS, and peritoneal metastases (P=0.004) and platelet-to-lymphocyte ratio (PLR) level (P=0.014) were independent prognostic predictors of OS according to Cox regression analysis. In second-line or posterior treatment, the number of metastatic sites (P=0.007), ECOG (P=0.011), and PLR level (P=0.033) were independent prognostic factors for PFS in AGC patients, and the number of metastatic sites (P=0.003), differentiation (P=0.030), and NLR level (P<0.001) were independent prognostic factors for OS according to Cox regression analysis.

**Conclusions:**

NLR, PLR, MLR, dNLR, and SII can reflect the short-term efficacy of immunotherapy in patients who underwent anti-PD-1 therapy with AGC. PLR is an independent prognostic factor for OS in AGC patients receiving first-line immunotherapy and PFS in those receiving second-line or posterior immunotherapy. And NLR was an independent prognostic factor for OS in AGC patients receiving second-line or posterior immunotherapy. The number of metastatic sites was significantly associated with the prognosis of AGC patients who received immunotherapy.

## Introduction

Gastric carcinoma is one of the most prevalent malignancies. It remains the fifth most common malignancy and the fourth leading cause of cancer-related death globally (1), and it is typically diagnosed at an advanced stage. Fluoropyrimidine-based regimens combined with platinum have been widely used as first-line chemotherapeutic agents for gastric cancer treatment (2). The application of trastuzumab benefited advanced HER2-positive gastric cancer patients (3).

Several programmed death 1/programmed cell death-legand1 (PD-1/PD-L1) pathway inhibitors have been approved for various tumor malignancies. Immuno-oncological agents targeting PD-1 have shown promising results against several cancer types, such as malignant melanoma (4), non-small cell lung cancer (5), and oesophageal carcinoma (6). According to the results of the ATTRACTION-2 trial (7), nivolumab significantly improved the survival of AGC patients who received two or more lines of therapy. CheckMate 649 trials showed that in patients with strong PD-L1 positivity (CPS≥5), nivolumab plus chemotherapy significantly improved overall survival (OS) and progression-free survival (PFS) in comparison with chemotherapy alone (8). Additionally, multiple anti-PD-1 therapy clinical trials are now underway or have been completed.

The introduction of immune checkpoint inhibitor (ICI) therapy has profoundly facilitated the development of therapeutic strategies for malignancies. However, for most gastric cancer patients, long-term efficacy cannot be maintained. Therefore, there is still the need for a reliable biomarker to find patients who derive a greater clinical benefit from immunotherapy. Previous research has suggested that gastric cancer with higher tumor mutation burden (TMB), high microsatellite instability (MSI-H), and Epstein-Barr virus (EBV)-positive status tends to prefer a therapeutic response to ICI therapy (9–11). However, a large number of patients benefit from a low tumor mutation burden, microsatellite stability (MSS), or EBV-negative state (12). Moreover, most biomarkers rely on tumor tissue testing, and the access and testing methods are complicated, which increases the invasive operation risk of patients. It is not easy to repeat testing. Therefore, convenient and straightforward inflammation indices as a part of prognostic cancer tools for AGC patients receiving anti-PD-1 therapy need to be explored and discovered.

Inflammation and inflammation-related factors are intricately linked to tumorigenesis (13, 14). Inflammatory factors have been suggested to be conducive to cancer initiation, progression, and metastasis (15, 16). Neutrophils are the most abundant white blood cells among circulating white blood cells and play a vital role in body immunity. In addition, neutrophils are also involved in the formation of the tumor microenvironment (17). Tumor‐associated neutrophils (TANs) are related to tumor development and progression without eliciting immunosuppressive activity (18). Our team’s previous findings suggested that extracellular traps released by neutrophils are also markers of AGC (19). As another critical cell in the immune system, lymphocytes may contribute to weakening the inhibitory effect of tumor cell proliferation (20). Furthermore, blood platelets play an essential role in cancer metastasis, and their mechanism in cancer is still unknown (21).

The neutrophil-to-lymphocyte ratio (NLR) and platelet-to-lymphocyte ratio (PLR) are markers that reflect the systemic inflammatory burdens in patients. Multiple studies have proven the advantages of NLR and PLR as tumor biomarkers in diagnosis, curative effect evaluation, and prognosis monitoring in gastric cancer (22), non-small cell lung cancer (23), and malignant melanoma (24). Therefore, they may serve as biomarkers in response to ICI therapy. Previous studies have shown that the derived neutrophil-to-lymphocyte ratio (dNLR) and NLR have similar prognostic values in various types of cancer (25). In addition, serum baseline dNLR levels among non-small cell lung cancer patients can predict the efficacy of immunotherapy (26). Furthermore, SII (the systemic immune-inflammation index) has advantages over NLR and PLR in predicting the survival of AGC patients (27). In immunotherapy, previous research reported that the SII could not serve as an independent factor of the prognosis of PFS in patients with gastric cancer (28). The monocyte-to-lymphocyte ratio (MLR) was shown to be an independent factor of prognosis for PFS and OS in a study involving both advanced gastric and colorectal cancer patients who accepted anti-PD-1 therapy (29). Overall, the prognostic value of inflammatory marker levels in patients with AGC remains contentious and imperfect. In this retrospective study, we analyzed the correlation between NLR, PLR, MLR, dNLR, SII, and PFS, OS to explore the changes in inflammatory markers and prognostic value in AGC patients treated with anti-PD-1. The main endpoints of this research are PFS and OS.

Hence, the clinical research sought to determine whether the NLR, PLR, MLR, dNLR, and SII can be used as markers for patients with AGC receiving immunotherapy. Concretely, our goals are as follows: i) to evaluate the association between the level of inflammatory markers and clinical characteristics. ii) Comparison of baseline, optimal effect, changes in inflammatory markers when progression, and the association between inflammatory markers and short-term effects. iii) To assess the relationship between the levels of inflammatory markers and the progression-free survival (PFS) and overall survival (OS) of patients with AGC. iv) To evaluate the correlation between the level of inflammatory markers and prognosis.

## Materials and Methods

### Patients and Data Collection

The retrospective analysis included data from one hundred and six patients with AGC treated with anti-PD-1 therapy between 20 July 2019 and 16 March 2021, which was enrolled by the Department of Medical Oncology and oncology-related departments, the First Affiliated Hospital of Anhui Medical University. Additionally, this project was approved by the Ethics Committee of the First Affiliated Hospital of Anhui Medical University. The clinical data were obtained from the electrical clinical medical record system, where variables including age, sex, differentiation, primary tumor location, metastatic site, number of metastatic sites, number of prior treatments, performance of the Eastern Cooperative Oncology Group, first-line chemotherapeutic regimen, alcohol consumption, serum tumor markers, HER2 status when starting anti-PD-1 therapy and previous therapies. The tumor-node-metastasis (TNM) stage was assessed based on the AJCC 8th edition TNM classification system. The inclusion criteria were as follows: i) all patients were diagnosed with gastric adenocarcinoma by pathology; ii) patients with advanced or unresectable cancer. iii) the patients’ physical condition can tolerate anti-PD-1 treatment or chemotherapy; iv) complete clinical data. The exclusion criteria were as follows: i) the patients’ cases were related to acute or uncontrolled infectious diseases, rheumatic disease, or severe cardiovascular or cerebrovascular diseases; ii) patients with two or more primary malignant carcinomas or other species of pathology; iii) when combined chemotherapy occurs if bone marrow suppression occurs, data after using granulocyte colony-stimulating factor or recombinant human thrombopoietin.

### Evaluation of the Inflammatory Markers

Patients underwent at least two cycles of immunotherapy, and peripheral blood samples were gathered up to two weeks before treatment. The samples were sonicated in a timely manner and centrifuged for 2 h. Venous blood (2 ml) was collected from the patient under fasting and resting states and injected into a vacuum anticoagulation tube containing dipotassium ethylenediaminetetraacetate (EDTA-K2). Additionally, 3-4 ml venous blood was collected and injected into a separation-promoting gel tube. Patients’ routine blood and blood biochemical parameters were collected. Peripheral blood samples of absolute neutrophil counts (ANCs), lymphocyte counts (ALCs), monocyte counts (AMCs), and platelet counts were recorded to calculate inflammatory markers. The NLR was calculated as ANC/ALC. The PLR was calculated as platelet count/ALC. The MLR was calculated as AMC/ALC. The dNLR was calculated as ANC/(WBC-ANC). The SII was calculated as ANC×platelet count/ALC. Inflammation markers were then categorized by Youden’s index using receiver operating characteristic (ROC) analysis as the cut-off.

### Follow-Up

Follow-up data were retrospectively obtained by the electrical clinical medical record system or telephone follow-up. To evaluate the therapeutic response, clinical evaluation and imaging examinations, including computed tomography (CT) scanning or other examinations needed, were performed every two courses or obvious clinical deterioration according to Response Evaluation Criteria in Solid Tumors (RECIST) version 1.1 or iRECIST. Clinical efficacy was evaluated by the researchers and defined as either complete response (CR), partial response (PR), stable disease(SD), or progressive disease (PD), on the basis of RECIST version 1.1 or iRECIST. For patients with PD who were evaluated for the first time, we reaffirmed the exclusion of false progression. The objective response rate (ORR) was calculated by CR plus PR. The disease control rate (DCR) was calculated as CR and PR and SD. PFS was calculated from the date of the initial treatment with the anti-PD-1 agent to disease progression or death or the last follow-up. OS was calculated from the date of the initial treatment with the anti-PD-1 agent to death, or the last follow-up. In the research, the date cut-off for follow-up was 1 December 2021. Treatment persisted until disease progression, unacceptable toxicity, or consent withdrawal.

### Statistical Analyses

Qualitative variables were compared between groups through the χ2 test or Fisher’s exact test. Inflammatory markers were then categorized by Youden’s index using ROC analysis as the cut-off value. Kaplan–Meier methodology was used to construct survival curves to estimate the PFS and OS of patients. Univariate and multivariate Cox proportional hazards regression of prognostic factors were conducted, and the 95% confidence interval (CI) was given. Multivariable models were generated based on including all variables with p<0.05 on univariable survival analysis. The final models were determined using stepwise backward method elimination of any variables with p<0.05 in the multivariable analyses. For all tests, a two-sided P<0.05 was considered statistically significant. All statistical analyses were performed using GraphPad Prism 8 (GraphPad Software, San Diego, CA) and SPSS version 22.0 (SPSS Inc., Chicago, IL, USA).

## Results

One hundred and six patients with AGC who underwent anti-PD-1 therapy were enrolled in this study, 53 patients in the first-line treatment, and 53 patients in the second-line or posterior line. The short-term effect was collected in all events. The number of PFS events was 81, 37 patients in first-line treatment and 44 patients in second-line or posterior treatment. And the number of OS events was 60, 27 patients in first-line treatment and 33 patients in second-line or posterior-line treatment. There were no unanticipated serious adverse events or other diseases during the study. Inflammatory markers were then categorized by Youden’s index using ROC analysis as the cut-off value [NLR=3.11 (range 0.53–12.93), PLR=172.79 (range 28.28–542.86), MLR=0.20 (range 0.01–1.26), dNLR=2.09 (range 0.29–6.28), SII=1140.91 (range 31.67–4687.76)]. The median PFS and OS time were 7.3 months (95% CI: 5.464–9.136) and 13.3 months (95% CI: 8.111–18.489) in first-line treatment, and 5.3 months (95% CI: 2.678–7.922) and 10.7 months (95% CI: 9.508–11.892) in the second-line or posterior line, respectively. The median follow-up durations in first-line treatment were 17.5 months (95% CI: 16.487–18.513) and 15.9 months (95% CI: 14.413–17.387) in the second-line or posterior line, respectively.

### Baseline Characteristics

The characteristics of the patients are given in **Table 1**. One hundred and six patients with AGC who accepted anti-PD-1 therapy were enrolled in this research, including 72 males (67.9%) and 34 females (32.1%). There were 29 patients (27.4%) aged >65 years and 77 (72.6%) aged ≤65 years; 53 patients (50.0%) received first-line treatment, and 53 (50.0%) received second-line or posterior therapy. Data were collected on the differentiation of gastric cancer in 101 patients and on the location of tumourigenesis in 102 patients. Data were also collected on 105 LDH levels, 96 CEA levels, 96 CA199 levels, and 79 CA724 levels. 7 patients were HER2 positive, 67 were negative and 32 had unknown HER2 status. Statistically, NLR level was found to correlate significantly with peritoneal metastasis (P=0.039) and CEA level (P=0.034). Patients with poor differentiation AGC (P=0.009) had a higher percentage of higher MLR levels in plasma. And in the dNLR groups, significant differences were found in peritoneal metastasis (P=0.005). Additionally, the number of metastatic sites was significantly related to NLR level (P=0.039), MLR level (P=0.031), dNLR level (P=0.005), and SII level (P=0.020). However, there were no relationships between inflammatory markers and other clinicopathologic factors.

**Table 1:** Baseline clinical characteristics of AGC patients.

	Total (n=106)	NLR≤3.11	NLR>3.11	P	PLR≤243.33	PLR>243.33	P	MLR≤0.20	MLR>0.20	P	dNLR≤2.09	dNLR>2.09		P	SII≤1140.91	SII>1140.91	P
	N (%)	(n=61)	(n=45)		(n=84)	(n=22)		(n=22)	(n=84)		(n=60)	(n=46)			(n=85)	(n=21)	
Age (years)																	
≤65	77 (72.6)	43	34	0.563	60	17	0.584	15	62	0.598	43	34		0.797	60	17	0.340
>65	29 (27.4)	18	11		24	5		7	22		17	12			25	4	
Gender																	
Male	72 (67.9)	43	29	0.510	57	15	0.977	15	57	0.977	43	29		0.346	59	13	0.509
Female	34 (32.1)	18	16		27	7		7	27		17	17			26	8	
Differentiation																	
Poor	61 (60.4)	32	29	0.079	49	12	0.785	8	53	0.009	33	28		0.180	48	13	0.427
Moderate or Well	40 (39.6)	28	12		33	7		14	26		27	13			34	6	
Location																	
Upper	35 (34.3)	20	15	0.084	27	8	0.586	10	25	0.582	20	15		0.318	28	7	0.772
Middle	21 (20.6)	9	12		17	4		3	18		9	12			16	5	
Low	34 (33.3)	25	9		30	4		6	28		23	11			29	5	
Others	12 (11.8)	5	7		9	3		3	9		6	6			9	3	
Metastatic site																	
Peritoneal metastasis																	
Negative	77 (72.6)	49	28	0.039	62	15	0.598	18	59	0.278	50	27		0.005	65	12	0.075
Positive	29 (27.4)	12	17		22	7		4	25		10	19			20	9	
Liver metastasis																	
Negative	64 (60.4)	36	28	0.739	51	13	0.890	14	50	0.726	35	29		0.623	49	15	0.248
Positive	42 (39.6)	25	17		33	9		8	34		25	17			36	6	
Lymph node metastasis																	
Negative	30 (28.3)	18	12	0.748	23	7	0.681	6	24	0.904	18	12		0.658	26	4	0.293
Positive	76 (71.7)	43	33		61	15		16	60		42	34			59	17	
Lung metastasis																	
Negative	90 (84.9)	53	37	0.507	70	20	0.515	19	71	>0.999	52	38		0.563	73	17	0.517
Positive	16 (15.1)	8	8		14	2		3	13		8	8			12	4	
Number of metastatic sites																	
≤2	77 (72.6)	49	28	0.039	63	14	0.287	20	57	0.031	50	27		0.005	66	11	0.020
≥3	29 (27.4)	12	17		21	8		2	27		10	19			19	10	
ECOG PS																	
0-1	99 (93.4)	59	40	0.132	77	22	0.340	21	78	>0.999	58	41		0.235	81	18	0.138
≥2	7 (6.6)	2	5		7	0		1	6		2	5			4	3	
First line chemotherapy																	
Fluorouracil +Platinum	65 (61.3)	40	25	0.426	50	15	0.695	14	51	0.571	41	24		0.146	51	14	0.773
Fluorouracil +Taxanes	14 (13.2)	6	8		11	3		4	10		5	9			11	3	
Others	27 (25.5)	15	12		23	4		4	23		14	13			23	4	
Response after chemotherapy																	
CR+PR	24 (22.6)	14	10	0.097	19	5	>0.999	4	20	0.155	16	8		0.055	21	3	0.206
SD	53 (50.0)	35	18		42	11		15	38		33	20			44	9	
PD	29 (27.4)	12	17		23	6		3	26		11		18		20	9	
Alcohol Consumption																	
Abstinence or low risk	94 (88.7)	54	40	0.953	73	21	0.453	20	74	>0.999	53		41	0.898	76	18	0.701
Hazardous or harmful	12 (11.3)	7	5		11	1		2	10		7		5		9	3	
Drugs																	
Camrelizumab	54 (50.9)	31	23	0.137	47	7	0.086	13	41	0.602	31		23	0.717	46	8	0.216
Sintilimab	37 (34.9)	22	15		27	10		7	30		21		16		28	9	
Toripalimab	5 (4.7)	5	0		4	1		1	4		4		1		5	0	
Pembrolizumab	7 (6.6)	2	5		5	2		0	7		3		4		4	3	
Nivolumab	3 (2.8)	1	2		1	2		1	2		1		2		2	1	
LDH																	
≤250	75 (71.4)	45	30	0.350	58	17	0.495	13	62	0.280	45		30	0.214	62	13	0.280
>250	30 (28.6)	15	15		25	5		8	22		14		16		22	8	
CEA																	
≤5	43 (44.8)	20	23	0.034	32	11	0.200	6	37	0.135	21		22	0.089	34	9	0.313
>5	53 (55.2)	36	17		45	8		14	39		35		18		46	7	
CA199																	
≤37	58 (60.4)	37	21	0.180	48	10	0.640	14	44	0.508	37		21	0.180	51	7	0.074
>37	38 (39.6)	19	19		30	8		7	31		19		19		28	10	
CA724																	
≤8.2	60 (75.9)	37	23	0.271	49	11	0.336	14	46	0.749	38		22	0.102	51	9	0.176
>8.2	19 (24.1)	9	10		13	6		3	16		8		11		13	6	
Molecular classification																	
HER negative	72 (67.9)	41	31	0.315	56	16	0.516	14	58	0.783	41		31	>0.999	55	17	0.086
HER positive	7 (6.6)	6	1		7	0		2	5		4		3		7	0	
Unknown	27 (25.5)	14	13		21	6		6	21		15		12		23	14	

### Relationships Between Inflammatory Markers and Short-Term Efficacy

**Figure 1:**
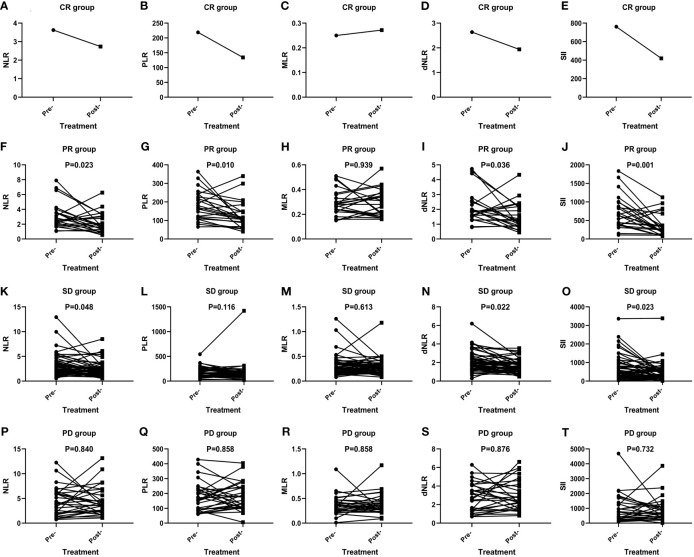
Efficacy of anti-PD-1 treatment according to the changes in inflammatory markers in AGC patients in the CR group **(A–E)**, PR group **(F–J)**, SD group **(K–O)**, and PD group **(P–T)**.

**Figure 2:**
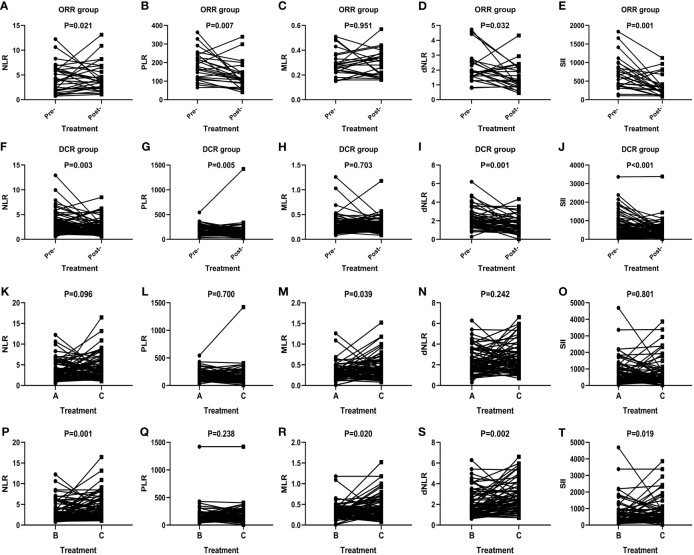
Efficacy of anti-PD-1 treatment according to the changes in inflammatory markers in AGC patients in the ORR group **(A–E)**, DCR group **(F–J)**, and subgroup analysis groups **(K–T)**. A: Levels of inflammatory markers in patients in the baseline. B: Levels of inflammatory markers in patients with optimal effects. C: Levels of inflammatory markers when disease progression.

The short-term effect of anti-PD-1 therapy treatment was collected in all events. These patients had received at least two courses of treatment when the outcome was achieved with PD-1 inhibitor therapy. One patient achieved CR, 23 patients achieved PR, 29 patients had PD, and 53 patients remained in stable condition. Additionally, the ORR and DCR were 22.6% and 72.6%. In the CR group, inflammatory markers, except for MLR, decreased after immunotherapy (**Figures 1A–E**). In particular, a significant decrease in SII was noticed among the PR (P=0.001) (**Figure 1J**), SD (P=0.023) (**Figure 1O**), ORR (P=0.001) (**Figure 2E**), and DCR (P<0.001) (**Figure 2J**) groups after anti-PD-1 therapy. A similar downward trend was also noticed in NLR with PR (P=0.023) (**Figure 1F**), SD (P=0.048) (**Figure 1K**), ORR (P=0.021) (**Figure 2A**), and DCR (P=0.003) (**Figure 2F**) and dNLR with PR (P=0.036) (**Figure 1I**), SD (P=0.022) (**Figure 1N**), ORR (P=0.032) (**Figure 2D**), and DCR (P=0.001) (**Figure 2I**). Additionally, a significant decline of PLR was also observed in PR (P=0.010) (**Figure 1G**), ORR (P=0.007) (**Figure 2B**), and DCR (P=0.005) (**Figure 2G**) after anti-PD-1 therapy. However, none of the levels of inflammatory markers in PD (**Figures 1P–T**) were significantly changed before and after anti-PD-1 therapy.

In total, inflammatory markers were recorded when anti-PD-1 treatment failed in 72 patients. Only MLR level increased significantly at the time of anti-PD-1 immunotherapy failure compared to baseline (P=0.039) (**Figure 2M**). However, statistically significant elevations in NLR (P=0.001) (**Figure 2P**), MLR (P=0.020) (**Figure 2R**), dNLR (P=0.002) (**Figure 2S**), and SII (P=0.019) (**Figure 2T**) were found in failure of anti-PD-1 treatment compared to optimal efficacy in AGC patients. No significant changes in inflammatory markers in other short-term efficacy groups after anti-PD-1 therapy (**Figures 1H, L, M** and **Figures 2C, H, K, L, N, O, Q**).

### Analysis of Kaplan–Meier Survival Curves

**Figure 3:**
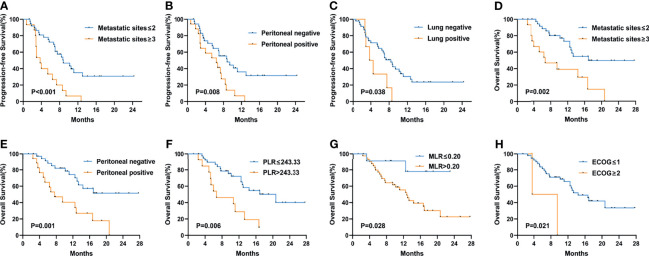
Kaplan–Meier curves of progression-free survival (PFS) and overall survival (OS) in the first-line treatment according to PFS: metastatic sites **(A)**, peritoneal metastasis **(B)**, lung metastasis **(C)**; OS: metastatic sites **(D)**, peritoneal metastasis **(E)**, PLR **(F)**, MLR **(G)**, and ECOG PS **(H)** at baseline. The P values were calculated using the log-rank test (two-sided).

**Figure 4:**
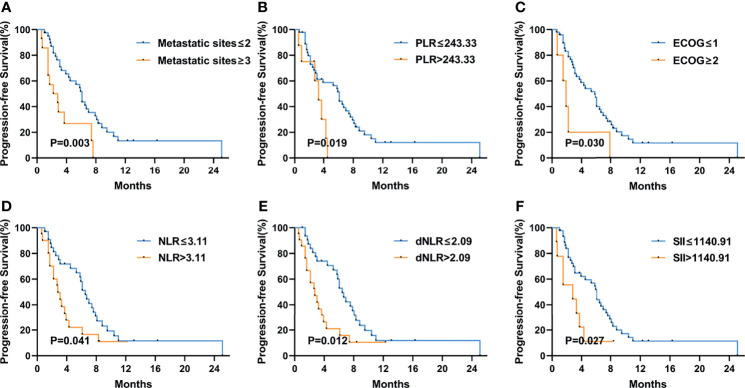
Kaplan–Meier curves of progression-free survival (PFS) in the second-line or posterior treatment according to metastatic sites **(A)**, PLR **(B)**, ECOG PS **(C)**, NLR **(D)**, dNLR **(E)**, and SII **(F)** at baseline. The P values were calculated using the log-rank test (two-sided).

**Figure 5:**
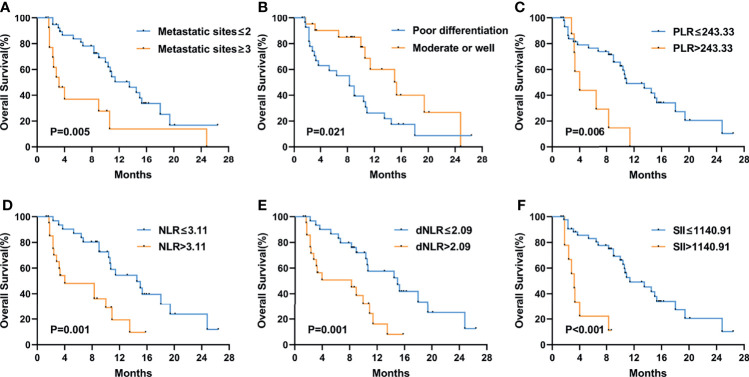
Kaplan–Meier curves of overall survival (OS) in the second-line or posterior treatment according to metastatic sites **(A)**, differentiation **(B)**, PLR **(C)**, NLR **(D)**, dNLR **(E)**, and SII **(F)** at baseline. The P values were calculated using the log-rank test (two-sided).

**Figure 3** shows the Kaplan–Meier curves of PFS and OS in the first-line therapy. The more than three metastatic sites group had a significantly shorter median PFS time than the less than three metastatic sites group (mPFS, 3.7 months, 95% CI: 2.564–4.836 vs. 8.8 months, 95% CI: 5.550–12.050, P<0.001) (**Figure 3A**). In comparison to the peritoneal metastasis negative group, the peritoneal metastasis positive group had a significantly poorer PFS time (mPFS, 6.4 months, 95% CI: 2.770–10.030 vs. 8.8 months, 95% CI: 5.011–12.589, P=0.008) (**Figure 3B**). Moreover, in contrast to the lung metastasis negative group, the lung metastasis positive group had a significantly poorer PFS time (mPFS, 3.7 months, 95% CI: 1.780–5.620 vs. 8.4 months, 95% CI: 6.272–10.528, P=0.038) (**Figure 3C**). Unfortunately, none of the inflammatory marker levels had significant differences in PFS in the first-line therapy. Similarly, more than three metastatic sites and positive peritoneal metastases had poorer OS compared to fewer than three metastatic sites and negative peritoneal metastases in the first-line therapy. (mOS, 6.7 months vs. 16.9 months, P=0.002) (**Figure 3D**), (mOS, 7.6 months, 95% CI: 3.621–11.579 vs. not reached, P=0.001) (**Figure 3E**), respectively. OS was shorter in patients with PLR>243.33 than in those with PLR ≤ 243.33 (mOS, 6.7 months, 95% CI: 0.963–12.437 vs. 20.7 months, 95% CI: 11.079–30.321, P=0.006) (**Figure 3F**). And compared to the MLR>0.20, patients with MLR ≤ 0.20 had a significantly longer OS (mOS, 12.8 months, 95% CI: 10.030–15.570 vs. not reached, P=0.028) (**Figure 3G**). Patients with low ECOG had a longer OS than those with high ECOG (mOS, 14.4 months vs. 3.6 months, P=0.021) (**Figure 3H**). **Figure 4** shows the Kaplan–Meier curves of PFS in second-line or posterior therapy. PFS was worse in the more than three metastatic sites group than in the less than three metastatic sites group (mPFS, 2.2 months, 95% CI: 0.183–4.217 vs. 6.1 months, 95% CI: 5.162–7.038, P=0.003) (**Figure 4A**). With respect to inflammatory makers, PFS was shorter in patients with PLR>243.33, NLR>3.11, dNLR>2.09, and SII>1140 than in those with PLR ≤ 243.33 (mPFS, 3.3 months, 95% CI: 2.070–4.530 vs. 6.1 months, 95% CI: 5.122–7.078, P=0.019) (**Figure 3B**); NLR ≤ 3.11 (mPFS, 2.8 months, 95% CI: 1.995–3.605 vs. 6.5 months, 95% CI: 5.457–7.543, P=0.041) (**Figure 3D**); dNLR ≤ 2.09 (mPFS, 2.8 months, 95% CI: 1.527–4.073 vs. 6.5 months, 95% CI: 5.367–7.633, P=0.012) (**Figure 3E**); and SII ≤ 1140 (mPFS, 2.8 months, 95% CI: 0.000–6.598 vs. 6.1 months, 95% CI: 5.151–7.049, P=0.027) (**Figure 3F**), respectively. Additionally, the low ECOG group had significantly longer PFS than the high ECOG group (mPFS, 5.8 months, 95% CI: 4.194–7.406 vs. 1.9 months, 95% CI: 1.041–2.759, P=0.030) (**Figure 4C**). **Figure 5** shows the Kaplan–Meier curves of OS in second-line or posterior therapy. OS was worse in the group with more than three metastatic sites than in the group with less than three metastatic sites (mOS, 3.2 months, 95% CI: 1.439–4.961 vs. 13.5 months, 95% CI: 9.214–17.786, P=0.005) (**Figure 5A**). And OS was worse in the poor differentiation group than in the moderate or well differentiation group (mOS, 8.3 months, 95% CI: 5.144–11.456 vs. 15.3 months, 95% CI: 9.721–20.879, P=0.021) (**Figure 5B**). Regarding inflammatory markers, high levels of PLR, NLR, dNLR, and SII are had shorter OS than those in low level groups (PLR: mOS, 4.0 months, 95% CI: 2.232–5.768 vs. 10.9 months, 95% CI: 6.369–15.431, P=0.006; NLR: mOS, 4.0 months, 95% CI: 0.000–8.916 vs. 14.5 months, 95% CI: 8.506–20.494, P=0.001; dNLR: mOS, 8.3 months, 95% CI: 1.174–15.426 vs. 15.0 months, 95% CI: 8.911–21.089, P=0.001; SII: mOS, 3.2 months, 95% CI: 2.031–4.369 vs. 11.4 months, 95% CI: 6.946–15.854, P<0.001) (**Figures 5C–F**), respectively.

### Prognostic Value of Inflammatory Markers in AGC Patients

For patients who accepted anti-PD-1 therapy in the first-line treatment, univariable and multivariate Cox regression analyses demonstrated only the number of metastatic sites (HR: 3.155, 95% CI: 1.601–6.216, P=0.001) was significantly correlated with PFS (**Table 2**). Nevertheless, no significant difference was observed in PFS within any other baseline clinical characteristic. In Addition, both PLR level (HR: 2.770, 95% CI: 1.234–6.219, P=0.014) and peritoneal metastases (HR: 3.098, 95% CI: 1.446–6.634, P=0.004) were independent prognostic predictors for GC patients with OS according to Cox regression analyses (**Table 3**). In second-line or posterior therapy, PLR level, ECOG PS, and number of metastatic sites were independent factors of prognosis for PFS in AGC patients depending on univariate and multivariate COX regression analysis (HR: 2.682, 95% CI: 1.083–6.639, P=0.033; HR: 3.595, 95% CI: 1.348–9.588, P=0.011; HR: 2.751, 95% CI: 1.324–5.714, P=0.007) (**Table 4**), respectively. With respect to OS in second-line or posterior therapy, patients with baseline NLR>3.11 had a shorter OS than those with NLR ≤ 3.11 (HR: 4.474, 95% CI: 1.942–10.308, P<0.001) (**Table 5**). The number of metastatic sites and differentiation were also independent factors of prognosis for OS in AGC patients (HR: 3.789, 95% CI: 1.590–9.028, P=0.003; HR: 0.398, 95% CI: 0.173–0.916, P=0.030) (**Table 5**).

**Table 2:** Univariate and multivariate analysis of factors according to PFS in AGC patients treated with anti-PD-1 drugs in the first-line therapy.

	Univariate analysis		Multivariate analysis	
	HR (95% CI)	P value	HR (95% CI)	P value
Age (years)				
≤65	1 (reference)			
>65	1.183 (0.513-2.728)	0.694	–	–
Gender				
Male	1 (reference)			
Female	0.964 (0.506-1.918)	0.964	–	–
Differentiation				
Poor	1 (reference)			
Moderate or well	0.731 (0.342-1.562)	0.416	–	–
Location		0.355		
Upper	1 (reference)			
Middle	2.225 (0.906-5.464)	0.081	–	–
Low	1.615 (0.680-3.839)	0.278	–	–
Others	1.356 (0.429-4.284)	0.603	–	–
Metastatic site				
Peritoneal metastasis				
Negative	1 (reference)		1 (reference)	
Positive	2.417 (1.235-4.731)	0.008	1.448 (0.638-3.287)	0.376
Liver metastasis				
Negative	1 (reference)			
Positive	1.714 (0.870-3.777)	0.116	–	–
Lymph node metastasis				
Negative	1 (reference)			
Positive	0.833 (0.378-1.835)	0.650	–	–
Lung metastasis				
Negative	1 (reference)		1 (reference)	
Positive	2.512 (1.016-6.209)	0.039	1.617 (0.633-4.131)	0.315
Number of metastatic sites				
≤2	1 (reference)		1 (reference)	
≥3	3.155 (1.601-6.216)	<0.001	3.155 (1.601-6.216)	0.001
ECOG PS				
0-1	1 (reference)			
≥2	2.901 (0.684-12.312)	0.130	–	–
First line chemotherapeutic regimen		0.364		
Fluorouracil+Platinum	1 (reference)			
Fluorouracil+Taxanes	1.142 (0.394-3.308)	0.807	–	–
Others	1.707 (0.809-3.602)	0.160	–	–
Alcohol Consumption				
Abstinence or low risk	1 (reference)			
Hazardous or harmful	1.097 (0.426-2.821)	0.848	–	–
NLR				
≤3.11	1 (reference)			
>3.11	1.627 (0.853-3.103)	0.136	–	–
PLR				
≤243.33	1 (reference)			
>243.33	1.987 (0.973-4.057)	0.055	–	–
MLR				
≤0.20	1 (reference)			
>0.20	1.958 (0.814-4.707)	0.126	–	–
dNLR				
≤2.09	1 (reference)			
>2.09	1.694 (0.888-3.232)	0.106	–	–
SII				
≤1140.91	1 (reference)			
>1140.91	1.812 (0.853-3.848)	0.117	–	–
LDH				
≤250	1 (reference)			
>250	1.167 (0.561-2.429)	0.679	–	–
CEA				
≤5	1 (reference)			
>5	0.746 (0.354-1.571)	0.439	–	–
CA199				
≤27	1 (reference)			
>27	1.048 (0.494-2.224)	0.903	–	–
CA724				
≤8.2	1 (reference)			
>8.2	0.858 (0.352-2.090)	0.736	–	–
Molecular classification		0.988		
HER negative	1 (reference)			
HER positive	0.973 (0.295-3.207)	0.965	–	–
Unknown	1.067 (0.441-2.582)	0.886	–	–

**Table 3:** Univariate and multivariate analysis of factors according to OS in AGC patients treated with anti-PD-1 drugs in the first-line therapy.

	Univariate analysis		Multivariate analysis	
	HR (95% CI)	P value	HR (95% CI)	P value
Age (years)				
≤65	1 (reference)			
>65	1.883 (0.786-4.513)	0.149	–	–
Gender				
Male	1 (reference)			
Female	0.985 (0.451-2.151)	0.969	–	–
Differentiation				
Poor	1 (reference)			
Moderate or well	0.553 (0.207-1.477)	0.231	–	–
Location		0.253		
Upper	1 (reference)			
Middle	2.652 (0.912-7.707)	0.073	–	–
Low	1.134 (0.410-3.134)	0.808	–	–
Others	1.238 (0.318-4.820)	0.758	–	–
Metastatic site				
Peritoneal metastasis				
Negative	1 (reference)		1 (reference)	
Positive	3.244 (1.517-6.936)	0.001	3.098 (1.446-6.634)	0.004
Liver metastasis				
Negative	1 (reference)			
Positive	1.486 (0.685-3.221)	0.313	–	–
Lymph node metastasis				
Negative	1 (reference)			
Positive	0.507 (0.220-1.171)	0.105	–	–
Lung metastasis				
Negative	1 (reference)			
Positive	1.163 (0.401-3.369)	0.781	–	–
Number of metastatic sites				
≤2	1 (reference)		1 (reference)	
≥3	3.192 (1.482-6.874)	0.002	1.296 (0.433-3.881)	0.644
ECOG PS				
0-1	1 (reference)		1 (reference)	
≥2	4.829 (1.092-21.350)	0.022	3.200 (0.643-15.925)	0.155
First line chemotherapeutic regimen		0.394		
Fluorouracil+Platinum	1 (reference)			
Fluorouracil+Taxanes	0.974 (0.222-4.265)	0.972	–	–
Others	1.775 (0.754-4.177)	0.189	–	–
Alcohol Consumption				
Abstinence or low risk	1 (reference)			
Hazardous or harmful	0.629 (0.187-2.108)	0.448	–	–
NLR				
≤3.11	1 (reference)			
>3.11	1.679 (0.778-3.625)	0.182	–	–
PLR				
≤243.33	1 (reference)		1 (reference)	
>243.33	2.959 (1.318-6.642)	0.006	2.770 (1.234-6.219)	0.014
MLR				
≤0.20	1 (reference)		1 (reference)	
>0.20	4.385 (1.036-18.569)	0.028	2.777 (0.626-12.311)	0.179
dNLR				
≤2.09	1 (reference)			
>2.09	1.781 (0.825-3.842)	0.136	–	–
SII				
≤1140.91	1 (reference)			
>1140.91	1.947 (0.873-4.346)	0.098	–	–
LDH				
≤250	1 (reference)			
>250	1.191 (0.520-2.726)	0.680	–	–
CEA				
≤5	1 (reference)			
>5	0.591 (0.235-1.487)	0.258	–	–
CA199				
≤27	1 (reference)			
>27	2.062 (0.836-5.083)	0.108	–	–
CA724				
≤8.2	1 (reference)			
>8.2	1.239 (0.449-3.419)	0.679	–	–
Molecular classification		0.813		
HER negative	1 (reference)			
HER positive	0.674 (0.157-2.900)	0.597	–	–
Unknown	1.173 (0.400-3.442)	0.771	–	–

**Table 4:** Univariate and multivariate analysis of factors according to PFS in AGC patients treated with anti-PD-1 drugs in the second-line or posterior therapy.

	Univariate analysis		Multivariate analysis	
	HR (95% CI)	P value	HR (95% CI)	P value
Age (years)				
≤65	1 (reference)			
>65	0.944 (0.492-1.814)	0.863	–	–
Gender				
Male	1 (reference)			
Female	1.227 (0.629-2.394)	0.547	–	–
Differentiation				
Poor	1 (reference)			
Moderate or well	0.924 (0.495-1.725)	0.805	–	–
Location		0.707		
Upper	1 (reference)			
Middle	1.272 (0.542-2.983)	0.581	–	–
Low	1.157 (0.532-2.515)	0.713	–	–
Others	1.741 (0.675-4.490)	0.251	–	–
Metastatic site				
Peritoneal metastasis				
Negative	1 (reference)			
Positive	1.293 (0.635-2.633)	0.477	–	–
Liver metastasis				
Negative	1 (reference)			
Positive	1.580 (0.847-2.947)	0.147	–	–
Lymph node metastasis				
Negative	1 (reference)			
Positive	1.231 (0.661-2.292)	0.512	–	–
Lung metastasis				
Negative	1 (reference)			
Positive	1.172 (0.558-2.461)	0.675	–	–
Number of metastatic sites				
≤2	1 (reference)		1 (reference)	
≥3	2.762 (1.358-5.618)	0.004	2.751 (1.324-5.714)	0.007
ECOG PS				
0-1	1 (reference)		1 (reference)	
≥2	2.712 (1.048-7.017)	0.032	3.595 (1.348-9.588)	0.011
First line chemotherapeutic regimen		0.558		
Fluorouracil+Platinum	1 (reference)			
Fluorouracil+Taxanes	1.007 (0.432-2.351)	0.987	–	–
Others	1.434 (0.723-2.846)	0.302	–	–
Alcohol Consumption				
Abstinence or low risk	1 (reference)			
Hazardous or harmful	0.938 (0.333-2.639)	0.904	–	–
NLR				
≤3.11	1 (reference)		1 (reference)	
>3.11	1.883 (1.009-3.514)	0.044	1.639 (0.809-3.319)	0.170
PLR				
≤243.33	1 (reference)		1 (reference)	
>243.33	2.716 (1.127-6.543)	0.020	2.682 (1.083-6.639)	0.033
MLR				
≤0.20	1 (reference)			
>0.20	1.497 (0.712-3.146)	0.284	–	–
dNLR				
≤2.09	1 (reference)		1 (reference)	
>2.09	2.168 (1.165-4.035)	0.012	1.268 (0.395-4.073)	0.690
SII				
≤1140.91	1 (reference)		1 (reference)	
>1140.91	2.356 (1.070-5.188)	0.028	0.765 (0.215-2.718)	0.679
LDH				
≤250	1 (reference)			
>250	1.288 (0.663-2.500)	0.454	–	–
CEA				
≤5	1 (reference)			
>5	1.879 (0.991-3.564)	0.050	–	–
CA199				
≤27	1 (reference)			
>27	1.038 (0.562-1.920)	0.904	–	–
CA724				
≤8.2	1 (reference)			
>8.2	2.087 (0.919-4.737)	0.072	–	–
Molecular classification		0.243		
HER negative	1 (reference)			
HER positive	1.448 (0.336-6.235)	0.619	–	–
Unknown	1.705 (0.907-3.207)	0.098	–	–

**Table 5:** Univariate and multivariate analysis of factors according to OS in AGC patients treated with anti-PD-1 drugs in the second-line or posterior therapy.

	Univariate analysis		Multivariate analysis	
	HR (95% CI)	P value	HR (95% CI)	P value
Age (years)				
≤65	1 (reference)			
>65	0.958 (0.453-2.026)	0.911	–	–
Gender				
Male	1 (reference)			
Female	1.575 (0.718-3.455)	0.254	–	–
Differentiation				
Poor	1 (reference)		1 (reference)	
Moderate or well	0.424 (0.200-0.901)	0.022	0.398 (0.173-0.916)	0.030
Location		0.348		
Upper	1 (reference)			
Middle	2.452 (0.905-6.649)	0.078	–	–
Low	1.540 (0.595-3.984)	0.373	–	–
Others	1.769 (0.555-5.643)	0.335	–	–
Metastatic site				
Peritoneal metastasis				
Negative	1 (reference)			
Positive	2.192 (0.970-4.954)	0.053	–	–
Liver metastasis				
Negative	1 (reference)			
Positive	0.617 (0.303-1.257)	0.180	–	–
Lymph node metastasis				
Negative	1 (reference)			
Positive	1.971 (0.943-4.119)	0.067	–	–
Lung metastasis				
Negative	1 (reference)			
Positive	0.494 (0.172-1.423)	0.183	–	–
Number of metastatic sites				
≤2	1 (reference)		1 (reference)	
≥3	2.792 (1.321-5.901)	0.005	3.789 (1.590-9.028)	0.003
ECOG PS				
0-1	1 (reference)			
≥2	2.652 (0.912-7.715)	0.063	–	–
First line chemotherapeutic regimen		0.449		
Fluorouracil+Platinum	1 (reference)			
Fluorouracil+Taxanes	1.659 (0.632-4.350)	0.304	–	–
Others	1.537 (0.681-3.469)	0.301	–	–
Alcohol Consumption				
Abstinence or low risk	1 (reference)			
Hazardous or harmful	1.252 (0.375-4.176)	0.714	–	–
NLR				
≤3.11	1 (reference)		1 (reference)	
>3.11	3.225 (1.542-6.744)	0.001	4.474 (1.942-10.308)	<0.001
PLR				
≤243.33	1 (reference)		1 (reference)	
>243.33	3.284 (1.345-8.019)	0.006	1.907 (0.670-5.423)	0.226
MLR				
≤0.20	1 (reference)			
>0.20	2.154 (0.824-5.631)	0.109	–	–
dNLR				
≤2.09	1 (reference)		1 (reference)	
>2.09	3.126 (1.493-6.545)	0.002	1.221 (0.289-5.162)	0.786
SII				
≤1140.91	1 (reference)		1 (reference)	
>1140.91	6.556 (2.527-17.009)	<0.001	1.409 (0.287-6.919)	0.673
LDH				
≤250	1 (reference)			
>250	1.374 (0.659-2.865)	0.395	–	–
CEA				
≤5	1 (reference)			
>5	1.373 (0.664-2.840)	0.390	–	–
CA199				
≤27	1 (reference)			
>27	1.662 (0.824-3.350)	0.151	–	–
CA724				
≤8.2	1 (reference)			
>8.2	1.367 (0.545-3.430)	0.503	–	–
Molecular classification		0.653		
HER negative	1 (reference)			
HER positive	1.039 (0.136-7.950)	0.971	–	–
Unknown	1.407 (0.676-2.927)	0.361	–	–

## Discussion

Although immunotherapy has been changing the landscape of oncologic therapies, there is still a lack of biomarkers that can assess therapeutic responses and predict prognosis. Previous studies reported that immunotherapy efficacy is related to HER2 expression, MMR state, EBV state, and PD-L1 expression (9–11). However, most biomarkers are too expensive and complicated for patients. Thus, convenient and straightforward inflammation markers need to be discovered. The tumorigenesis and development of cancers are closely related to inflammation (13). Inflammatory markers have been reported to reflect the biological characteristics of AGC (30). The NLR, PLR, MLR, dNLR, and SII are prognostic markers that have been used to predict the outcome of immunotherapy in non-small cell lung cancer (31) and renal cancer (32). However, there is little information about the application of inflammatory markers in patients with AGC receiving anti-PD-1 treatment.

In our study, none of the inflammation markers corresponded precisely to clinicopathologic factors. We found that peritoneal dissemination was associated with high levels of NLR and dNLR in patients with AGC at baseline. It is generally known that the peritoneum is the most frequent metastatic site for AGC, which often predicts poor prognosis (33). Similarly, Patients with more than three metastatic sites are related to high levels of NLR, MLR, dNLR, and SII, which is comparable to the results of previous studies (22). The outcome indirectly suggested that high levels of inflammatory markers might be associated with poor prognosis in AGC patients. Additionally, a high MLR is related to poor differentiation. At present, the MLR has been reported as a marker in advanced gastric and colorectal cancer patients treated with ICIs for the efficacy of DCR and an independent prognostic factor (29).

Although changes in inflammatory markers can reflect the patients’ response to treatment (34), few studies have reported dynamic changes in inflammatory markers in patients with AGC who underwent anti-PD-1 treatment. In our research, we discovered that inflammatory markers were closely associated with short-term efficacy in patients with AGC accepting anti-PD-1 treatment. Although only one patient achieved CR, four inflammatory markers declined after immunotherapy. This change also happened in the PR and SD groups. In the PR group, we found that NLR, PLR, dNLR, and SII levels decreased significantly in patients who achieved the optimal effect. In the SD state, this trend was also observed among NLR, dNLR, and SII. Additionally, similar downward trends were also noticed in ORR and DCR groups with NLR, PLR, dNLR, and SII. In particular, significant decreases in NLR, dNLR, and SII were observed among the PR, SD, ORR, and DCR groups after anti-PD-1 therapy. The outcome suggested that inflammation markers, especially SII, may be involved in the associated short-term efficacy. The SII appears to have more advantages than the NLR, PLR, and other inflammatory markers in AGC patients accepting anti-PD-1 therapy in predicting short-term efficacy, which might be because the SII combines several blood cell counts, reduces errors, and can reflect the balance between host inflammation and the immune response more comprehensively and objectively. Similarly, a significant decrease in SII was also observed after neoadjuvant chemotherapy in patients with gastric cancer (30). Notably, the bone marrow toxicity of neutrophils may change due to chemotherapy, which may affect the results. In our study, a significant decline in inflammatory markers in the PR and SD groups did not appear in the PD group. This suggested that the decline in inflammatory markers might be based on changes in short-term efficacy without interference from bone marrow suppression.

To our knowledge, our research is the first to show that AGC patients who underwent anti-PD-1 therapy with a higher baseline SII level had worse short-term effects than patients who had a lower SII level. However, SII, which has a curative effect evaluation value in the short-term curative effect, did not show statistical significance in PFS or OS according to multivariate Cox regression analyses. This situation may be due to many factors influencing the conversion of recent tumor control to prolonged survival and the limited sample size. Additionally, a significant decrease was also noticed in the PR, SD, ORR, and DCR groups with NLR and dNLR. These results are in accordance with previous reports that enrolled patients with both advanced gastric and colorectal cancer (29). To further explore the changes in inflammatory markers in PD patients, we compared the levels of inflammatory markers in patients receiving immunotherapy with baseline and disease progression and with optimal efficacy and disease progression. Interestingly, baseline inflammatory marker levels in AGC patients all tended to rise when immunotherapy failed, but only MLR was statistically significantly elevated. Furthermore, NLR, MLR, dNLR, and SII were significantly increased in AGC patients during disease progression compared to optimal efficacy, which echoes the results of a previous study exploring changes in NLR before and after immunotherapy (35). Taken together, the outcomes suggested that NLR, MLR, dNLR, as well as SII, can predict the progression of the disease for patients receiving anti-PD-1 treatment. The dynamic changes in inflammatory markers may imply the impact of anti-PD-1 therapy on the immune system.

In this research, the baseline levels of inflammatory markers were not related to PFS in the first-line therapy. This situation is associated with many factors that might affect the prognosis and survival of patients receiving first-line therapy. However, the prognosis of patients with fewer than three metastatic sites significantly outperformed that of patients with more than three metastatic sites, according to the multivariate Cox analysis. Additionally, both peritoneal metastasis positive and high PLR level were independent factors of prognosis for short OS in AGC patients by multivariate Cox regression analyses. Interestingly, previous clinical trials have reported that the outcome of AGC patients receiving PD-1 inhibitor monotherapy with peritoneal metastasis negative or fewer metastatic sites is significantly better than those of patients with peritoneal metastasis positive or more metastatic sites (36). Our study obtained similar results according to peritoneal metastasis positive and more metastatic sites for poorer prognosis. In second-line or posterior therapy, the number of metastatic sites, ECOG PS, and PLR level were independent factors of prognosis for PFS in AGC patients by multivariate Cox proportional hazards regression. It is well known that patients with poor ECOG status often do not tolerate conventional doses of medication and have poorer outcomes and prognosis. Similar to our findings, in a prospective analysis, ECOG≥2 was a poor prognostic factor for AGC patients on second-line chemotherapy (37). In terms of OS, a retrospective study that included 1733 patients with progressive gastric cancer, NLR and histological differentiation were reported to be independent prognostic factors for fluoropyrimidine-platinum combination chemotherapy after first-line treatment in patients with advanced gastric cancer (38). We obtained similar results in AGC patients treated with anti-PD-1 in the second-line or posterior therapy. And patients with fewer metastatic sites had significantly better OS than those with more metastatic sites. However, in our study, HER2 status was not an independent factor affecting the prognosis of patients with progressive gastric cancer receiving immunotherapy, which may be related to the fact that immunotherapy significantly improved the prognosis of patients with HER2-negative gastric cancer, compensating for the pre-existing survival differences across HER2 status. Notably, previous reports indicated that the PLR can serve as an independent prognostic factor for patients receiving ICI treatment in non-small cell lung (23) and malignant melanoma (39), which corresponded to our results. To the best of our knowledge, this is the first report that a high baseline PLR is an independent predictor of poor PFS in the second-line or posterior therapy and poor OS in first-line therapy in AGC patients receiving anti-PD-1 therapy, which further enriches the application value of inflammatory markers in immunotherapy.

At present, the potential mechanism of PLR in anti-PD-1 therapy is not fully understood. As studies have shown, malignant tumors are often accompanied by platelet rise and aggregation. Reducing the number of platelets or inhibiting the function of platelets inhibits the metastasis of tumor cells (21). The reasons for this phenomenon are multifocal. First, platelets can promote the formation of tumor thrombi and protect tumor cells from the blood flow shear force of blood flow and NK cell lysis (40). Second, platelets have been identified as a major source of basic fibroblast growth factor (bFGF) and transforming growth factor-β (TGF-β), promoting tumor growth (41). Third, platelets can enhance the invasion potential of tumor cells. Studies have shown that circulating tumors (CTCs) activate platelet secretion of TGF-β, which induces platelets to accelerate or maintain CTC epithelial-mesenchymal transition (EMT) to promote abscission, migration, and invasion of tumor cells from the primary site (42). Blocking the TGF-β signaling pathway might attenuate tumor extravasation and pulmonary metastasis (43). Platelets bound by tumor cells release soluble media, such as ADP, thromboxane A2 (TXA2), or high mobility group 1 (HMGB1), which are linked to Toll-like receptor 4 (TLR4) on platelets and mediate platelet-tumor cell interactions, which increase the permeability of the blood vessel wall, thereby promoting the metastasis of tumor cells (44). Additionally, platelets also play an essential role in promoting the adhesion, retention, and metastasis of cancer cells in blood vessels, and current studies have shown that the molecules that mediate platelet adhesion function are mainly p-selectin and integrin αIIBβ3 expressed on the surface of activated platelets. Blocking p-selectin or αIIBβ3 integrin with antibodies significantly reduces platelet interactions with tumor cells (45). In summary, a higher PLR could serve as an indicator of poorer clinical outcomes in anti-PD-1 therapy, which provides new insights for future research.

Nevertheless, this study is limited by being a single-center retrospective study, which might result in selection biases and confounders. Thus, further prospective studies are required to support the use of inflammatory markers as prognostic factors for AGC patients treated with anti-PD-1 therapy. Consequently, there is still a larger sample size and an extended follow‐up to confirm the conclusions.

## Conclusions

In summary, peritoneal dissemination was significantly associated with high levels of NLR and dNLR in patients with AGC at baseline. Additionally, more than three metastatic sites was more frequent in AGC patients accepting anti-PD-1 therapy with high levels of NLR, MLR, dNLR, and SII. The decrease in NLR, PLR, MLR, dNLR, and SII levels was associated with better short-term efficacy of immunotherapy. For patients with optimal efficacy, elevated NLR, MLR, dNLR, and SII levels often indicate disease progression. And elevated baseline MLR level is also indicative of disease progression. High PLR level is a poor independent prognostic factor that affects PFS in the second-line or posterior therapy and OS in the first-line therapy with AGC patients receiving immunotherapy. Furthermore, NLR and histological differentiation were independent prognostic factors for OS in the second-line or posterior therapy. The number of metastatic sites and peritoneal metastases were significantly associated with the prognosis of AGC patients who received immunotherapy. Alternatively, ECOG≥2 is a poor prognostic factor for PFS in AGC patients receiving second-line or posterior immunotherapy. Our findings can further screen AGC patients who benefit from immunotherapy and are useful for treatment decisions in clinical practice.

## Data Availability Statement

The original contributions presented in the study are included in the article/supplementary material. Further inquiries can be directed to the corresponding authors.

## Ethics Statement 

The studies involving human participants were reviewed and approved by the Ethics Committee of the First Affiliated Hospital of Anhui Medical University. The patients/participants provided their written informed consent to participate in this study.

## Author Contributions

KG and YZ planned and designed the study. ZQ, QW, and HW collected and analyzed the data and wrote the article. YJ, ML, WW, and YL helped to collect data. ZZ and TZ helped to analyze the data. All authors read and approved the final version.

## Funding

This study was supported by grants from the Natural Science Foundation of Anhui Province (No. 1908085QH333), the Key Research and Development Project of Anhui Province (No. 202004j07020044), and the Natural Science Research Project of Anhui Provincial University (No. KJ2018ZD019).

## Conflict of Interest

The authors declare that the research was conducted in the absence of any commercial or financial relationships that could be construed as a potential conflict of interest.

## Publisher’s Note

All claims expressed in this article are solely those of the authors and do not necessarily represent those of their affiliated organizations, or those of the publisher, the editors and the reviewers. Any product that may be evaluated in this article, or claim that may be made by its manufacturer, is not guaranteed or endorsed by the publisher.
